# Haploid identification in maize

**DOI:** 10.3389/fpls.2024.1378421

**Published:** 2024-04-19

**Authors:** Abil Dermail, Mariah Mitchell, Tyler Foster, Mercy Fakude, Yu-Ru Chen, Khundej Suriharn, Ursula Karolina Frei, Thomas Lübberstedt

**Affiliations:** ^1^ Department of Agronomy, Faculty of Agriculture, Khon Kaen University, Khon Kaen, Thailand; ^2^ Department of Agronomy, Iowa State University, Ames, IA, United States; ^3^ Plant Breeding Research Center for Sustainable Agriculture, Faculty of Agriculture, Khon Kaen University, Khon Kaen, Thailand

**Keywords:** maize hybrid breeding, doubled haploid, haploid selection, haploid verification, automated sorting

## Abstract

Doubled haploid (DH) line production through *in vivo* maternal haploid induction is widely adopted in maize breeding programs. The established protocol for DH production includes four steps namely *in vivo* maternal haploid induction, haploid identification, genome doubling of haploid, and self-fertilization of doubled haploids. Since modern haploid inducers still produce relatively small portion of haploids among undesirable hybrid kernels, haploid identification is typically laborious, costly, and time-consuming, making this step the second foremost in the DH technique. This manuscript reviews numerous methods for haploid identification from different approaches including the innate differences in haploids and diploids, biomarkers integrated in haploid inducers, and automated seed sorting. The phenotypic differentiation, genetic basis, advantages, and limitations of each biomarker system are highlighted. Several approaches of automated seed sorting from different research groups are also discussed regarding the platform or instrument used, sorting time, accuracy, advantages, limitations, and challenges before they go through commercialization. The past haploid selection was focusing on finding the distinguishable marker systems with the key to effectiveness. The current haploid selection is adopting multiple reliable biomarker systems with the key to efficiency while seeking the possibility for automation. Fully automated high-throughput haploid sorting would be promising in near future with the key to robustness with retaining the feasible level of accuracy. The system that can meet between three major constraints (time, workforce, and budget) and the sorting scale would be the best option.

## Introduction

Hybrid cultivars are common in maize as they produce stable and high yields. Traditional methods for developing homozygous inbred lines as parental lines of hybrids require six to eight generations to fully benefit from heterosis (Hallauer et al., 2010). The timeline might be extended for several more years since further yield trials of hybrids have to be accomplished prior to varietal release. Doubled haploid (DH) technology reduces the time constraint by rapid development of fully homozygous inbred lines within two generations only. Thus, it enhances the genetic gain per unit time and supports requirements for varietal registration such as distinctness, uniformity, and stability (Geiger and Gordillo, 2009).

*In vitro* and *in vivo* methods are available to produce DH lines. *In vitro* methods use anther or microspore culture to develop haploids, while *in vivo* methods use maternal or paternal haploid induction (Geiger and Gordillo, 2009; Seguí-Simarro et al., 2021). *In vitro* methods have shown strong genotype dependency in maize, which makes it unfeasible for routine use in maize breeding programs. As a result, *in vivo* haploid induction is commonly used in the development of maize DH lines today, given its reliability and efficiency for large-scale production of DH lines (Chaikam et al., 2019). In general, the production of DH lines via *in vivo* maternal induction system involves four general steps: (1) induction of haploid seed, (2) identification of haploids, (3) haploid genome doubling, and (4) self-pollination of doubled haploids. The details of these steps have been reviewed by Chaikam et al. (2019) and Maqbool et al. (2020). For the first step, maternal haploid induction requires elite haploid inducers. Specific male genotypes are needed with the capability to generate haploid seed in induction crosses with donor germplasm. Elite haploid inducers with high haploid induction rate and adaptation to local environments are suggested to improve the efficiency of DH technology (Prasanna, 2012). A list of available maize haploid inducers with different haploid induction rates was included in Liu et al. (2016). In addition, breeding strategies for developing maize haploid inducers have been reviewed by Trentin et al. (2020).

**Figure 1:**
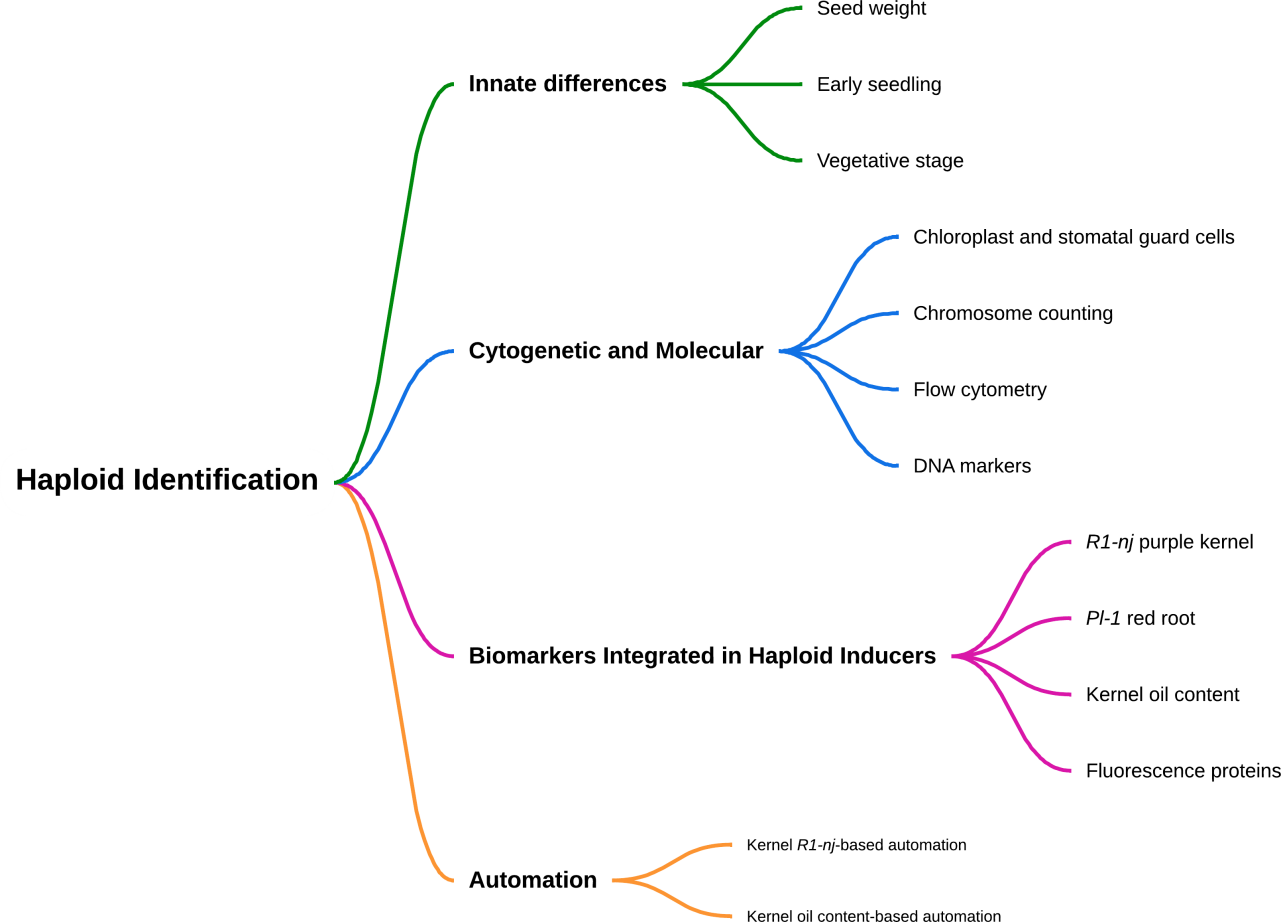
The schematic way of different methods of haploid identification in maize.

To date, the highest haploid frequencies of modern haploid inducers across donor populations range from 10 to 15% (Liu et al., 2016), indicating that up to 75 haploids could be obtained from roughly 500 seeds of typical F_1_ or F_2_ donor ears. Since those inducers still produce a relatively small fraction of haploid seed, haploid identification is critically important. Classical techniques like determination of stomata size and density, chromosome counting, and flow cytometry are accurate but laborious, costly, and time-consuming. Genetic markers like *R1-nj* mediated kernel and embryo coloration (Nanda and Chase, 1966) are embedded in most haploid inducers to facilitate visual haploid sorting at the kernel stage. However, misclassification due to false discovery or false negatives must be considered. Other biomarkers including *Pl-1* mediated red roots (Rotarenco et al., 2010), kernel oil content (Melchinger et al., 2013), and transgenic markers (Yu and Birchler, 2016), were introduced to improve the accuracy of haploid selection (**Figure 1**). This review (i) summarizes available methods for haploid induction in major cereal crops, including maize, (ii) provides background on innate differences between haploids and diploids, (iii) summarizes available methods for haploid identification in maize including cytological and molecular methods, biomarkers integrated in haploid inducers including transgenic markers, and (iv) gives an outlook for the perspective of automated haploid sorting.

## Haploid induction

**Figure 2:**
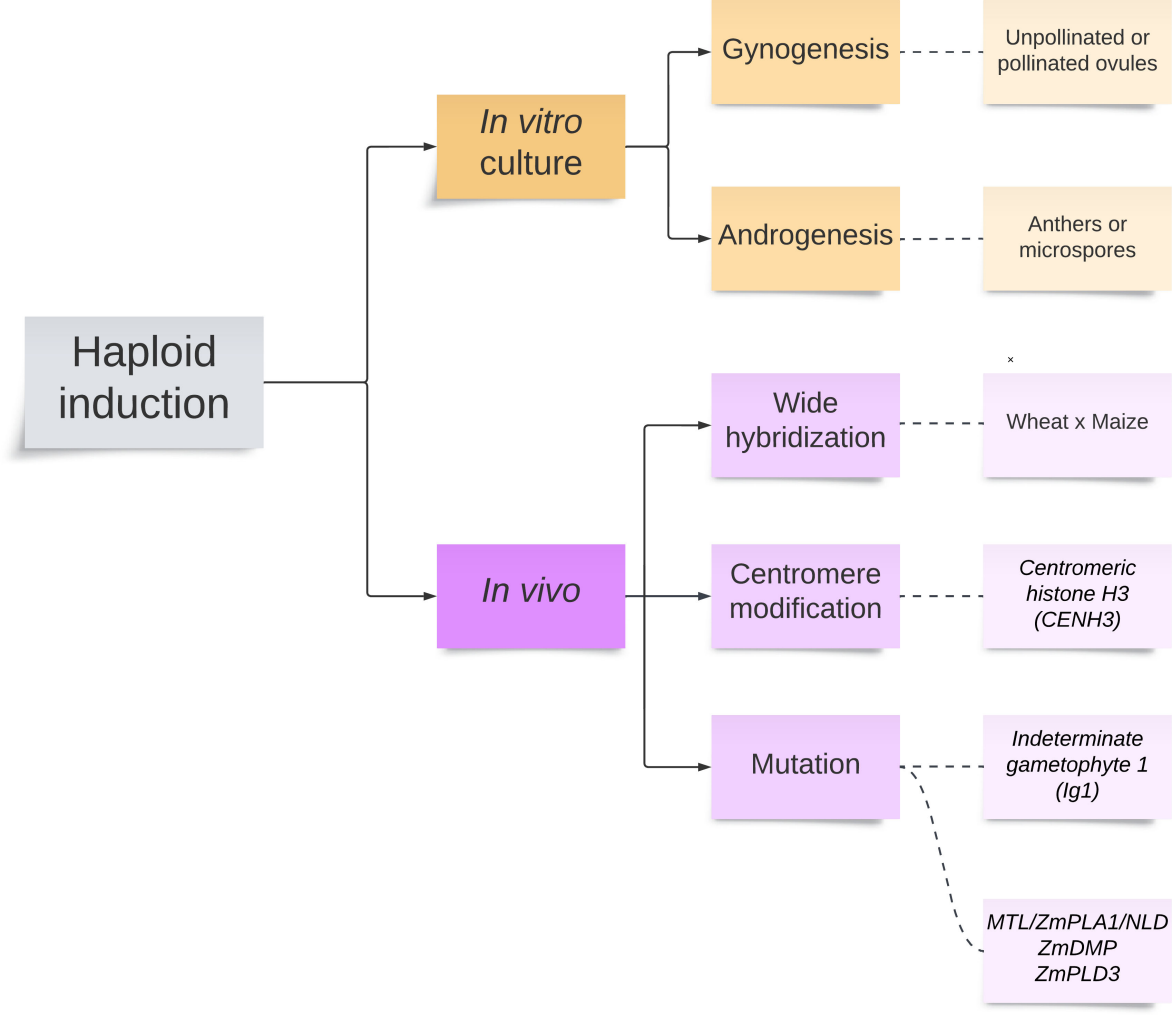
Haploid induction in major cereal crop species, including *in vitro* and *in vivo* methods.

Two major methods to produce haploids in major cereal crops are available, including *in vitro* tissue culture and *in vivo* genetic induction (**Figure 2**). *In vivo* genetic induction can be accomplished through wide hybridization, centromere modification via *centromeric histone H3* (*CENH3*), and mutation via *Indeterminate gametophyte 1* (*Ig1*)-mediated paternal system and *MTL/ZmPLA1/NLD* and *ZmDMP*-mediated maternal system. All methods discussed in this section should be able to induce maize haploids, except for the wide hybridization.

### *In vitro* culture

*In vitro* culture has two approaches for creating haploids: gynogenesis and androgenesis. Gynogenesis involves culturing either unpollinated or pollinated ovules, while androgenesis involves culturing anthers or microspores. *In vitro* gynogenesis is more effective for crops where anther culture and wide hybridization are not possible (Forster et al., 2007). However, lower embryo production rates have limited its practical use in crop breeding (Tang et al., 2006). In contrast, *in vitro* androgenesis is more favorable for inducing haploids in maize (Zheng et al., 2003). The complete protocol to induce maize haploids by *in vitro* culture of pollinated ovaries (gynogenesis) can be found in Tang et al. (2006). Meanwhile, the procedure for haploid induction via *in vitro* culture of microspores (androgenesis) is available in Zheng et al. (2003). Although attempts have been made (Niazian and Shariatpanahi, 2020), strong genotype dependence and a high possibility of mutations during the culture process make *in vitro* culture challenging for maize. The other limitations include the need for special skills for accurate anther staging, anther pretreatment, and media preparation. Further protocol developments are encouraged, emphasizing temperature pretreatments of donor materials, media and nutrient composition, and broader evaluation of genotypes and species (Hale et al., 2022).

Critical factors determining the success of *in vitro* culture during the induction and regeneration phases include plant genotype, microenvironment, composition of culture medium, the developmental stage of initial gametophytic cells, physical treatments of cultured gametophytic cells, and application of different additives and plant growth regulators (Niazian and Shariatpanahi, 2020). They mentioned plant growth regulators such as chlormequat, polyamines (putrescine, spermidine, and spermine), stress hormones (abscisic acid, jasmonic acid, salicylic acid), DNA demethylating agents, histone deacetylase inhibitors, cellular antioxidants, cell wall remodeling agents (arabinogalactan-proteins), and compatible solutes (proline and chitosan) as activator for enhancing the frequency of haploid induction.

### Wide hybridization

Wide hybridization is utilized to produce haploids in some crop species (Ishii et al., 2016). This process involves the selective elimination of chromosomes of one parent, resulting in haploid embryos. The endosperm often fails to proliferate and sustain embryo development, making embryo rescue following wide hybridization essential to recover haploids. The selective elimination of chromosomes of one parent in wide hybridization experiments is due to several hypotheses, including differences in the timing of cell cycles between two parents, parent-specific inactivation of centromeres, asynchrony in nucleoprotein synthesis, and degradation of chromosomes by host-specific nucleases (Watts et al., 2018). Wheat breeding programs commonly induce haploids by wide hybridization between wheat and maize. The maize chromosomes are eliminated during the early development of the hybrid seeds after wheat spikes were pollinated with maize pollen (Niu et al., 2014). The efficient protocol for haploid production in durum and common wheat via wide hybridization with maize is available in Niu et al. (2014). Guan et al. (2024) proposed the Wheat × Maize System for producing wheat doubled haploid via maize pollen. This technique has room for improvement in terms of production efficiency, and many factors such as genotype, environment, and treatments can influence it. Optimizing maize genotype selection and haploid plantlet doubling treatment can improve DH production efficiency. The establishment of an industry-scale production procedure for the Wheat × Maize System can meet the demands of scientific research on wheat genetics and breeding and wheat production.

## Centromere modification

Centromeres plays a critical role in the process of proper chromosome segregation during cell division. The centromeric histone H3 (CENH3) protein specifies the identity of the centromere, and lack of *CENH3* leads to cell death due to improper distribution of chromosomes (Ravi and Chan, 2010). *CENH3*-mediated haploid induction approach has been developed, which involves substituting the native *CENH3* gene function with a chimeric *CENH3* gene construct, resulting in haploid embryos. This approach has been successfully demonstrated in *A. thaliana* and maize. Kelliher et al. (2016) developed haploid inducer lines, CENH3−/− and CENH3:RNAi complemented by AcGREEN-tailswap-CENH3 or AcGREEN-CENH3 transgenes. The complemented CENH3−/− lines produced low rate of gynogenic haploid induction (3.6%) when backcrossed as males. These results demonstrate that *CENH3*-tailswap transgenes can be used to engineer *in vivo* haploid induction systems into maize plants.

Recent work is ongoing to explore the potential of centromere-mediated genome elimination for haploid induction in maize. Non-transgenic modifications to CENH3 have been reported to induce maize haploids. When CENH3 from closely related plant species complements a *cenh3* null, it produces plants that are fertile but induce haploids on crossing by CENH3 wild type plants. It implies that the introgression of alien CENH3 may produce non-transgenic haploid inducers (Britt and Kuppu, 2016). Wang et al. (2021) demonstrated a simplified method regarding crossing maize lines that are heterozygous for a *cenh3* null mutation to induce haploids. They noticed that crossing +/*cenh3* to wild-type plants in both directions can generate haploid progeny. The *cenh3* genotype of the gametophyte determines genome elimination, indicating that centromere failure is caused by CENH3 dilution during the postmeiotic cell divisions prior to gamete formation. The *cenh3* haploid inducer works as a vigorous hybrid and can be transferred to other lines in a single cross, making it versatile for multi-purpose.

### *Indeterminate gametophyte1*-mediated paternal system

The *ig1* (*indeterminate gametophyte*) is responsible for inducing paternal haploids in maize. It was first discovered in the Wisconsin-23 (W23) line and is located on chromosome 3 (Kermicle, 1994). The *ig1* mutant induces irregularities in seed formation that increase the rate of haploid induction in progenies (Kermicle, 1971). When the *ig1/ig1* homozygous mutant is used as the female parent and wild type is used as the male parent, chromosomes from the *ig1* mutant are eliminated, resulting in a paternal wild-type haploid that contains the *ig1* mutant cytoplasm. The induction of paternal haploids is influenced by parental genetic background and the constitution of donor genotype cytoplasm, rather than the specific *ig* gene in the male gametophyte (Kermicle, 1969; Geiger, 2009). In addition, the low frequency of haploids makes the *ig* gene less attractive for commercial scale haploid induction in maize. However, the *ig1/ig1* genetic stock can be used to convert inbred lines to their cytoplasmic male sterile (CMS) version (Geiger, 2009).

### MTL/ZmPLA1/NLD, ZmDMP, and ZmPLD3-mediated maternal system

Maternal haploid production involves haploid inducers as male parents to pollinate source germplasm as female parents. The efficiency of maternal haploid production depends on the availability of inducer genotypes with high haploid induction rate (HIR). To fully take advantage of this technology, the average HIR of modern haploid inducers should exceed 10% (Hu et al., 2016).

Several studies have detected the major QTL *qhir1* located on chromosome 1 with different haploid inducers from different source germplasm. A major locus *ggi1* on chromosome 1 (bin 1.04) with the closest SSR umc1169, controlling *in situ* gynogenesis, has also been identified (Barret et al., 2008). They suggested that the occurrence of a partial failure of the male inducer leading to segregation distortion at the *ggi1* locus associated with gynogenetic induction with incomplete penetrance. Prigge et al. (2012) identified two major QTL, namely *qhir1* and *qhir8*, located on chromosome 1 (bin 1.04) and 9 (bin 9.01), respectively, and six minor QTL, namely *qhir2*, *qhir3*, *qhir4*, *qhir5*, *qhir6*, and *qhir7*. They proposed that *qhir1* is a key factor to stimulate HIR, and the number of QTL and QTL effects obtained for HIR varied depending on populations and generations.

The haploid inducibility is a result of a genetic interaction between the paternal haploid inducer and the maternal donor genotype (Lashermes and Beckert, 1988). Two QTL for haploid inducibility, namely *qmhir1* and *qmhir2*, were detected on chromosome 1 (bin 1.01) and 3 (bin 3.08/09), respectively, with partially dominant effects. The maternal QTL *qmhir1* flanked by SSR marker loci umc1292 and bnlg1014 accounted for 14.70% of the phenotypic variance, and *qmhir2* flanked by SSR marker loci umc1844 and umc2277 contributed 8.42% to the phenotypic variance (Wu et al., 2014).

To date, three genes have been successfully cloned and become transferable to improve HIR: 1) MATRILINEAL (MTL)/ZEA MAYS PHOSPHOLIPASE A1 (ZmPLA1)/NOT LIKE DAD (NLD) (Gilles et al., 2017; Kelliher et al., 2017; Liu et al., 2017), 2) ZmDMP (Zhong et al., 2019), and 3) ZmPLD3 (Li et al., 2021). The first cloned gene, MTL/ZmPLA1/NLD, is located within QTL *qhir1*, while the second cloned gene, ZmDMP, is located within QTL *qhir8* (Prigge et al., 2012). It is important to note that these three cloned genes have the same subcellular localization, specifically expressed in membranes of sperm cells, and act as synergistic effects. It means that haploid induction will be triggered if the MTL gene is present, and the HIR will significantly increase when the other two genes (ZmDMP and/or ZmPLD3) are present. Therefore, future breeding of haploid inducers for enhanced HIR is feasible by accumulating as many favorable alleles responsible for HIR as possible.

Two hypotheses have been proposed to explain the biological mechanism of haploid induction in maize: 1) single fertilization and 2) selective elimination of inducer chromosomes in early embryo development post-fertilization. Comprehensive reviews on these two hypotheses can be found in Trentin et al. (2020). Regarding the first hypothesis, single fertilization occurs when only the egg or the central cell are fertilized, resulting in kernels with defective endosperms or with haploid embryos, respectively (Sarkar and Coe, 1966). Regarding the second hypothesis, evidence for the process of selective elimination of inducer chromosomes from embryonic cells has been reported by Zhao et al. (2013). They found that most inducer chromosomes were eliminated from haploid embryonic cells within a week of pollination and from endosperm cells of defective kernels by 15 days after pollination.

## Innate differences between haploids and diploids

**Figure 3:**
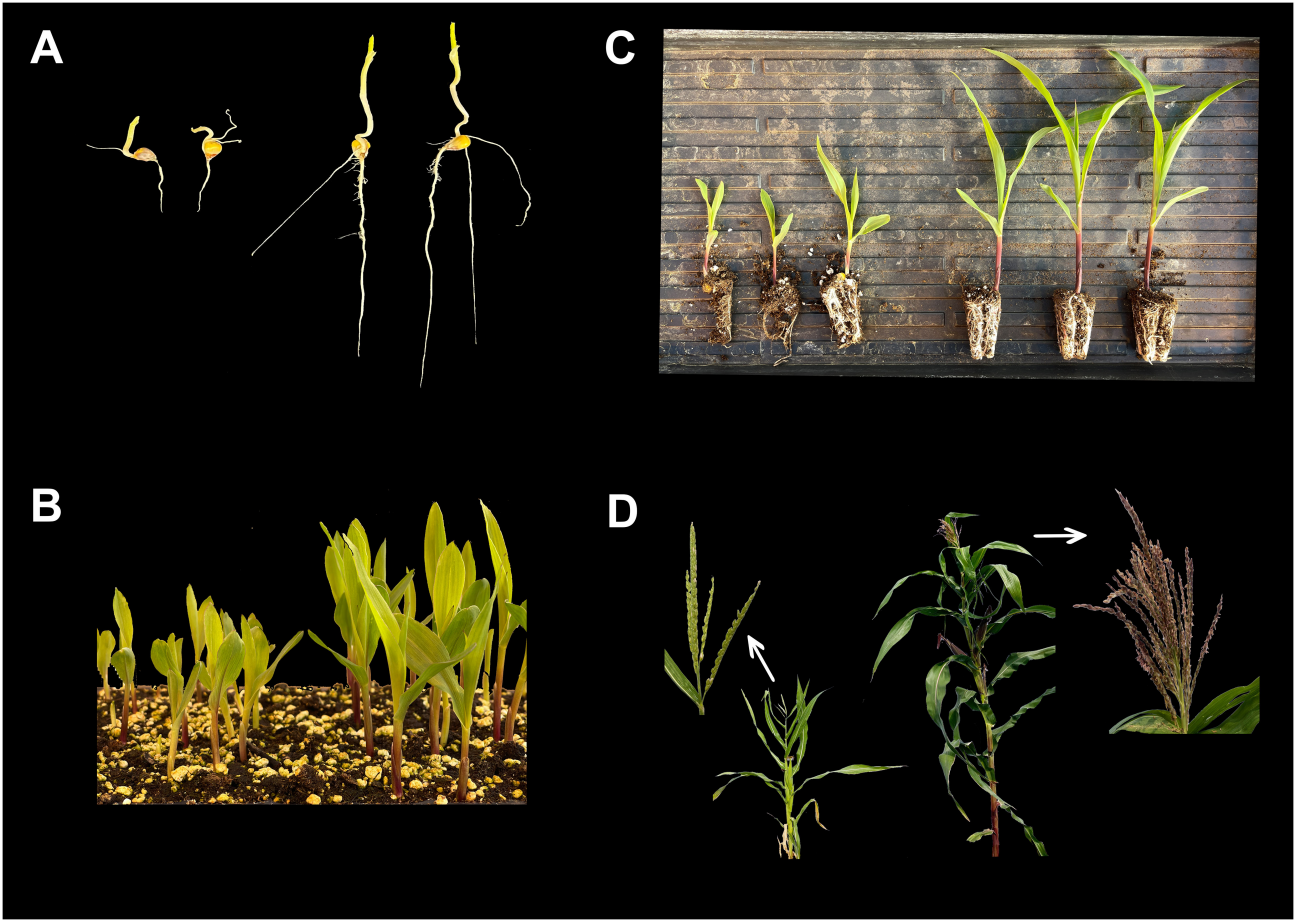
The innate differences between haploid (left) and diploid (right) individuals in maize at early seedling or 4 days after sowing **(A)**, seedling in plug trays or 7-8 days after sowing **(B)**, V2/V3 seedling or 10-14 days after sowing **(C)**, and adult stages **(D)**. 1A: Haploids show shorter coleoptile and radicle than diploids. 1B: Haploid seedlings show shorter leaf blades and plant height than diploid seedlings. 1C: Haploid seedlings show shorter leaf blades and plant height and lower root density than diploid seedlings. 1D: Adult haploid plants produce tassels without anthers and pollen, while adult diploid plants produce tassels with fresh anthers and fertile pollen. Besides, haploid plants show lower vigor with narrow, erect, pale or light green leaves. Diploids show better vigor with dark green leaves and purple stem.

Innate differences between haploid and diploid individuals in maize were studied by Chase (1964). Mature haploid plants showed a 30% shorter plant stature, 24% smaller internode diameter, 23% shorter leaf length, 44% narrower leaf area, and only 65% biomass weight compared to isogenic diploid plants. Haploid plants had fewer leaves, an earlier tasseling date, a reduced number of tassel branches, prolific ears with a reduced number of ear rows and spikelets per row than diploid plants (Chase, 1964, 1969). The majority of haploid individuals exhibited complete male sterility, yet the female gametophyte was partially fertile (Chalyk, 1994). Maize studies on haploid/diploid characterization rely on reduced vigor throughout maize development from seedlings to mature plants (**Figure 3**) and are discussed below. However, for haploid identification in induction crosses, haploid seed is compared with diploids that result from crosses with the inducer, thus hybrid seed. This means, in addition to the ploidy effects described by Chase (1964), it is possible to exploit the heterosis effect in addition to the ploidy effect, when differentiating haploid – diploid seed or seedlings in induction crosses. Differences should thus be even more pronounced than described in this section.

### Seed weight

Haploid and diploid seeds were shown to differ in seed weight (SW), with haploid seed weighing less on average than diploid seed (Smelser et al., 2015). However, SW distributions of haploid and diploid seed overlap, depending on donor genotypes (Smelser et al., 2015). Thus, deploying a threshold across and even within populations with different genetic backgrounds is challenging to effectively differentiate haploid and diploid seed. The weight of single maize kernels can vary from 130-150 mg in small-seeded popcorn up to 636 mg in Giant Peruvian or Cuzco white maize (Rooney and Suhendro, 2001; Serna-Saldívar, 2010). When implementing SW for haploid – diploid discrimination, it is advisable to estimate the range of SW for a subset of each unique population by excluding haploid kernels in the upper weight distribution and including more diploid kernels in the lower weight distribution before deploying an automated process (Smelser et al., 2015).

### Early seedling

Quantifiable differences between diploid and haploid young seedlings have been identified for radical length (RL), coleoptile length (CL), and the number of lateral seminal roots (NLSR). After 96 h of germination, haploid individuals tend to have shorter RL and CL and lower NLSR than equivalent diploids. Other properties of haploids such as thin coleoptiles and radicles and low number of root hairs can also be noticed (Chaikam et al., 2015). This method may be helpful to serve as an independent marker system when donor populations are not amenable to use of *R1-nj* markers due to complete inhibition of *C1-l* gene or masking of natural purple pigmentation. This reduced false positive detection up to 6-fold in certain donor populations as compared to *R1-nj* marker alone (Chaikam et al., 2015). Previous studies showed the effectiveness of combining *R1-nj* and seedling traits. Battistelli et al. (2013) used visual selection among putative haploid seedlings for reduced vigor 72 h after germination in water-soaked germination papers to remove likely false positives before performing genome doubling.

The generation cycle can be further shortened up to six weeks by bypassing seed dormancy via embryo culture to allow early haploid selection based on primary root length (PRL) and daily growth rate (GR) (Vanous et al., 2019). In this study, haploid and diploid individuals started showing differentiation two days after germination when haploids have significantly shorter PRL and slower GR. This approach was most accurate and practical on the third day of germination via support vector machine learning with a 2% false negative rate (FNR) and 9% false positive detection rate (FDR) or the fourth day of germination via regular observations with 3% FNR and 11% FDR (Vanous et al., 2019).

Those two methods are beneficial within the timeline of DH production since the colchicine induced haploid genome doubling is usually carried out 3-4 days after germination via the submersion method (Chaikam and Mahuku, 2012) or 10-12 days after planting via the injection method (Vanous et al., 2017). Both methods save resources including colchicine treatment costs and greenhouse space, since false positives would be discarded before haploid genome doubling process (Baleroni et al., 2021).

However, handling thousands of seedlings to achieve the targeted number of haploids is laborious. Another drawback is the need to estimate specific thresholds for each donor genotype by performing a training set for each targeted population because of the significant interaction between donor and ploidy level for RL, CL, and NLSR, making this step more time consuming (Baleroni et al., 2021). A significant number of undetermined seedlings that could not be classified into either haploids or diploids, from 11.8% to 17.8%, through this method has been reported when different donor populations were used (Greenblatt and Bock, 1967; Chaikam et al., 2015). If undetermined seedlings are discarded, additional induction crosses are required to compensate for potential haploid losses.

### Vegetative stage

There are many visual cues to aid in distinguishing haploids and diploids during the life cycle’s vegetative phase. First, haploid plants are less vigorous and are significantly shorter in height (Liu et al., 2020). In addition, maize diploid plants resulting from induction crosses with *R-nj* inducers have red/purple coloration within the stalk while haploid plants lack purple coloration although genetic background may also be involved (Chaikam et al., 2015). Finally, maize haploid plants have narrow, erect leaves (Melchinger et al., 2013). Sekiya et al. (2020) applied this screening method in super sweet corn donor populations at V_2_/V_3_ stage or 10-12 days after sowing (DAS) under greenhouse condition. The inducer population PI4001 was used to generate haploids. All putative haploid seedlings with either green color of the first leaf sheath or albino phenotypes were confirmed to be true haploids with flow cytometry. Meanwhile, diploids showed purple first leaf sheath. Using this method, they could significantly reduce false positives of haploid selection based on *R1-nj* seed marker ranging from 84% to 98% among super sweet corn populations (Sekiya et al., 2020). In contrast, Ribeiro et al. (2020) found that this method was not reliable when assuming short plants with lower leaf sheath as putative haploids at 8 days after sowing (DAS) since all putative haploids obtained with those criteria were confirmed to be true diploids regarding flow cytometry. For rogueing of potential false positives in haploid nurseries, it is advisable to perform multiple plant inspections with different selection intensities from two weeks after transplanting to early reproductive stage, as these visual characteristics are most noticeable near the V_6_ vegetative phase of growth (Aboobucker et al., 2022).

### Cytogenetic and molecular methods

**Figure 4:**
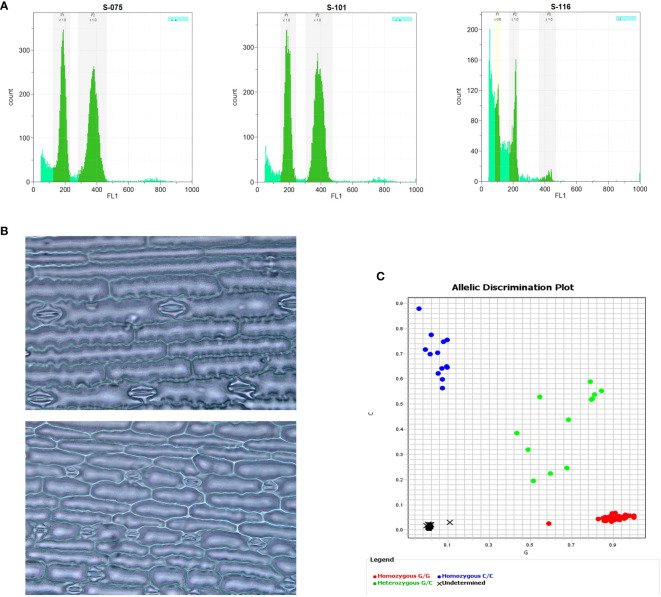
Cytological and molecular methods in haploid identification via flow cytometry **(A)**, stomatal guard cell length **(B)**, and SNP marker **(C)**. 2A: The first peak value (G1) of haploid nuclei has half channel numbers as much as the diploid’s G1, representing 100 vs. 200 for haploid and diploid, respectively. 2B: Haploid (upper) has shorter length but higher density of stomatal guard cells than diploid (lower). 2C: Haploids have zero copies of the *mtl* allele and two copies of the maternal *MTL* allele (homozygous G/G, red), while diploids have one copy of the *mtl* allele and one copy of the *MTL* allele (heterozygous G/C, green).

Cytological and molecular procedures for determining ploidy levels in maize can be performed in several ways including inspection of chloroplast and stomatal guard cell properties, chromosome counting, flow cytometry, and DNA marker analyses. Although those methods were shown to be reliable (**Figure 4**), each of them has limitations regarding time, labor, equipment requirements, and costs (**Table 1**).

**Table 1:** List of cytogenetic methods for ploidy identification between haploid (H) and diploid (D) in maize, along with their principal differentiation, the levels of workload, and the ranges of values between haploid and diploid.

Method	Principal differentiation	Levels of workload				Values (H vs. D)
		Time	Labor	Equipment	Cost	
Chloroplast number per stomata (CN)	Haploid has lower CN than diploid	Moderate	Low	Low	Low	2.54 vs. 3.60 ^1^ 2.41 vs. 3.47 ^2^ 2.50 vs. 3.40 ^3^
Stomatal guard cell length (SGCL)	Haploid has shorter SGCL than diploid	Moderate	Low	Low	Low	30.00 μm vs. 40.00 μm ^4^ 37.36 μm vs. 48.93 μm ^5^ 146.30 μm vs. 199.19 μm ^6^ 28.29 μm vs. 38.13 μm ^7^
Chromosome count	Haploid has only one set of the whole genomes	High	High	Low	Low	10 vs. 20 ^8^
Flow cytometry	The peak value (G1) of haploid nuclei has half channel numbers as much as the diploid’s G1	Low	Low	High	High	25 C vs. 50 C ^9^ 50 C vs. 100 C ^10,11,12^ 100 C vs. 200 C ^13,14,15^ 200 C vs. 400 C ^16,17^ 190-210 C vs. 390-410 C ^18^
DNA marker	Haploid shows one band or allele from maternal donor, while diploid carries heterozygous bands or alleles from both maternal donor (D) and paternal inducer (I)	Low	Low	Moderate	Moderate	SSR loci: − bnlg2259: 200 bp (D) vs. 180 bp (I) ^19^ − bnlg2082: 250 bp (D) vs. 200 bp (I) ^19^ − chr1-275.3: 400 bp (D) vs. 500 bp (I) ^17^ − chr7-20.7: 400 bp (D) vs. 600 bp (I) ^17^

^1^ Ho et al. (1990); ^2^ Wan et al. (1992); ^3^ Beaumont and Widholm (1993); ^4^ Sarkar and Coe (1966); ^5^ Choe et al. (2012); ^6^ Molenaar et al. (2019); ^7^ da Silva et al. (2020); ^8^ Kiesselbach and Petersen (1925); ^9^ Liu et al. (2017); ^10^ Wan et al. (1991); ^11^ Ismaili and Mohammadi (2016); ^12^ Jiang et al. (2021); ^13^ Couto et al. (2013); ^14^ Couto et al. (2015); ^15^ Baleroni et al. (2021); ^16^ Rádi et al. (2020); ^17^ Li et al. (2021); ^18^ Kelliher et al. (2017); ^19^ Dong et al. (2018).

### Chloroplast and stomatal guard cell properties

In many plant species, associations have been found with cell or organ size to ploidy level. For example, in tall fescue (*Festuca arundinacea*, Schreb.) an increase in ploidy level indicated an increase in stomatal size with a decrease in overall stomatal density (Byrne et al., 1981). Comparable results were found across different species of orchids (Orchidaceae) where increasing plant ploidy levels lead to an increase in guard cell size (Miguel and Leonhardt, 2011). In maize, the stomatal size of haploids was smaller and denser (Randolph, 1932). Corzo (1952) found that the number of stomata per m^2^ ranged from 32.93 to 38.21 in diploids and from 42.75 to 61.99 in haploids.

The number of chloroplasts (CN) in stomatal guard cells positively correlates with the ploidy levels in many crop species (Butterfass, 1973). In maize, however, the gap of CN across ploidy levels is different, with the tetraploid/diploid ratio being much higher than the diploid/haploid ratio. The tetraploid/diploid ratio ranges from 1.91-1.97 while the diploid/haploid ratio ranges from 1.36-1.44 across maize genotypes (Ho et al., 1990; Beaumont and Widholm, 1993). Although this method is rapid and practical, the small differences of CN between diploid and haploid could be biased when plants are grown under different light conditions. Under low light interception, both chlorophyll content and the number of CN in maize were reduced (Ren et al., 2017). Thus, the use of CN for ploidy differentiation is more convenient in polyploid crops such as potato (Alsahlany et al., 2019; Kramer and Bamberg, 2019) and watermelon (Noh et al., 2012; Zhang et al., 2019).

Stomatal guard cell length (SGCL) can determine maize ploidy levels that haploids have lower SGCL than diploids (Sarkar and Coe, 1966; Choe et al., 2012; Molenaar et al., 2019; da Silva et al., 2020). The smaller SGCL size found in haploids might be explained by the lower amount of DNA (Lomax et al., 2009) and the lower gene dosage of three signal epidermal patterning factors (EPFs) controlling stomatal development (Hunt and Gray, 2009). Despite the possibility to distinguish haploids, within group variation for haploid, doubled haploid, and heterozygous SGCL was found (Molenaar et al., 2019). The strong overlap of distributions of SGCL between haploids and diploids may be due to SPATIal effects or the fluctuations of sunlight when plants are grown in the greenhouse. Increasing the sampling number of stomata per leaf per plant, using light-controlled chamber, and maintaining the stomatal turgor pressure by submerging the roots using flood irrigation tables were proposed to reduce the individual plant variance (Molenaar et al., 2019). In addition to overlap issues, SGCL varied across germplasm source, leaf stage, and ploidy level. Thus, it is important to determine the threshold for each leaf stage of each donor genotype prior to haploid selection in diverse and large populations. This method is practical, cost-saving, yet not suitable for high throughput. The whole procedure of measuring SGCL was described by Kim et al. (2010).

### Chromosome counting

Counting chromosomes under the microscope is a direct method and accurate to identify ploidy levels in most plant species (Doležel et al., 2007). In maize, haploids have 10 chromosomes while diploids have 20 chromosomes (Kiesselbach and Petersen, 1925). Three general steps in chromosome counting (CC) include (1) material collection and pretreatment, (2) material fixation, and (3) staining and flattening of chromosomes. Specific chemicals and protocol modifications are required depending on the plant species, chromosome sizes, genotypes, and tissue sources (Dhooghe et al., 2011). The materials could be cells undergoing either mitosis or meiosis. Mitotic CC commonly works well on root tip cells from youNG seedlings. However, other meristematic cells from young floral buds, leaves or callus can also be used (Maluszynska, 2003). In contrast, meiotic CC utilizes pollen mother cells, and the complete procedure can be found in Windham et al. (2020).

However, some constraints with CC do exist. Tissues containing cells under mitotic division may not always be available (Doležel and Bartoš, 2005). The needs of plant-specific enzymatic treatments are error-prone when preparing the metaphase C slides (Ribeiro et al., 2020) and counting many small chromosomes (Dhooghe et al., 2011) hinder the possibility for high-throughput cell analyses. Most obviously, CC is extremely laborious, time consuming and requires trained scientists (Doležel and Bartoš, 2005). Perhaps this method is more convenient in *in vitro* DH production where operators are famiIiar with CYTological skills. Studies on maize haploid plants derived from the anther culture, for example, determined the ploidy level via mitotic CC (Tsay et al., 1986; Wan et al., 1989). Above-mentioned difficulties and lack of young scientists well trained in adopting CC (Goldblatt, 2007) contribute to a significant shift from CC to flow cytometry.

### Flow cytometry

Flow cytometry (FC) has many beneficial features. First, it is a rapid and accurate method to determine the ploidy status among plant species in early growth stages (De Laat et al., 1987), taking only 3.5 min per sample (Molenaar et al., 2019). Second, it is highly sensitive to detect DNA content variation within maize species (Rayburn et al., 1989) and to distinguish maize plants with different ploidy levels from haploid, diploid, mixoploid, aneuploid to polyploid (Cousin et al., 2009; Rádi et al., 2020; Jiang et al., 2021). Third, it is useful to determine cell cycle status (Brown et al., 1991), DNA contents among plant species (Galbraith et al., 1983), plant genome size (Yanpaisan et al., 1999), the level of generative polyploidy, nuclear replication state, and endopolyploidy (polysomaty) (Doležel et al., 2007). Those advantages resulted in FC analysis being the gold standard classification method to verify true haploid individuals and to estimate false positive rates in maize (Melchinger et al., 2016).

Steps of the FC method include preparing nuclei suspensions, staining nuclei suspensions, analyzing them using a flow cytometer, and determining nuclei ploidy (Cousin et al., 2009). Regarding preparing the nuclei sample, recent progress has been made from razor chopping method (Galbraith et al., 1983), bead-beating method (Roberts, 2007), multichannel pipetting (Cousin et al., 2009), and crushing devices (Erich Pollähne GmbH, 2019). The movement from manual single-tube to high-throughput allows robust analysis from 12 samples per operator (Galbraith and Lambert, 2012) up to 1,000 samples per operator per day (Erich Pollähne GmbH, 2019). In the step of staining nuclei, DNA-sPEcific fluorescent dye is used, for instance, fluorochromes DAPI (Rayburn et al., 1989) and propidium iodide (Arumuganathan and Earle, 1991). The nuclei are then passed through a flow cytometer-cell sorter (Rayburn et al., 1989). Since the amount of fluorescence emitted from each nucleus reflects its DNA content, and its DNA content is correlated to its ploidy levels, ploidy can be determined by the fluorescence intensity of stained cell nuclei isolated from plant tissue (Bohanec, 2003). The peak value (G1) of each sample nuclei is commonly set to 50 channels (1C) and 100 channels (2C) for haploid and diploid, respectively (Jiang et al., 2021). The full protocol of FC through plant single-cell suspensions can be found in Rayburn et al. (1989) and Arumuganathan and Earle (1991), while the procedure of high throughput FC method is available in Cousin et al. (2009).

In maize, FC can fit in ploidy determination of samples derived from both *in vitro* through microspore culture and *in vivo* haploid induction methods. While the typical source of nuclei in *in vitro* method is either callus tissue (Wan et al., 1991, 1992) or early developmental stage of plantlets (Dwivedi et al., 2015; Ismaili and Mohammadi, 2016), the sample nuclei in *in vivo* method are derived from meristematic organs of young seedlings such as leaves (Molenaar et al., 2019; Baleroni et al., 2021) and root tips (Rádi et al., 2020). However, nuclear DNA content variation exists, even within species (Arumuganathan and Earle, 1991; Rayburn et al., 1993). Therefore, careful selection of control genotypes is crucial for establishing accurate 1C and 2C DNA peaks. It is common in maize haploid selection that the position of the G1 peak of each sample is compared with that of the female parent or donor genotype assigned as known diploid control (Rádi et al., 2020; Baleroni et al., 2021).

### DNA markers

DNA molecular markers can be classified in two groups: (1) dominant markers such as amplified fragment length polymorphism (AFLP) and random amplified polymorphic DNA (RAPD) and (2) codominant markers such as simple sequence repeat (SSR) and single nucleotide polymorphism (SNP). Codominant markers are preferable since heterozygotes (F_1_ diploids) can be distinguished from homozygotes (donor female), allowing the determination of haploids and diploids. Therefore, this section focusses on the practical use of both SSR and SNP markers in ploidy determination in maize.

Microsatellites or single sequence repeats (SSRs) are repetitive DNA sequences in which each locus is individually amplified with PCR (Xu, 2010). The advantages of microsatellites are single locus, multi-allelic, transferable across populations, highly polymorphic and informative, and randomly distributed throughout eukaryotic genomes. However, it is susceptible to mutation, difficult to automate, and time consuming for primer preparation (Acquaah, 2009). To ensure the reliability of SSRs in ploidy determination, it is important to first screen primers showing polymorphism of the parents. Then, polymorphic markers can be employed in the progenies to discriminate homozygous and heterozygous alleles regarding different band sizes. Haploids carry only one band from the maternal donor, while diploids carry heterozygous bands from both the maternal donor and paternal inducer. Currently, multiple SSR loci are available and can be accessed from the MaizeGDB database (http://www.maizegdb.org/ssr.php).

The following are illustrations of assaying SSR markers for haploid selection. Battistelli et al. (2013) found 5 of 25 SSR primers which are polymorphic between donor hybrid 30/31 and inducer KEMS. Thus, those five markers (bnlg1175, bmc1714, bnlg1520, bnlg1233, bnlg1258) were applied to determine the ploidy of the progenies. One of those markers, bnlg1233, was also reliable to detect maternal haploids in other studies (Couto et al., 2015; Ribeiro et al., 2020). Li et al. (2009) screened 300 SSR primers using donor ZD958 and inducer CAUHOI as checks and obtained 40 polymorphic markers distributed evenly across ten chromosomes. Zhao et al. (2013) screened 84 SSR primers and found only three polymorphic markers (X10, X35, umc1317) between donor Z58 and inducer CAU5. Belicuas et al. (2007) assayed 122 SSR primers for polymorphism between donor hybrid BRS1010 and inducer line W23 and selected two markers (mmc0021 and mmc0081) for further androgenetic haploid identification. Zhang et al. (2008) screened 28 SSR primers and found two polymorphic markers (umc2059 and bnlg1065) between donor Hua24 and inducer HZI1. Using the same inducer line with different donor genotypes, Qiu et al. (2014) assayed 100 SSR primers and found several markers performing clear polymorphic bands, for example, umc1747, umc1784, bnlg1909, umc1870, bnlg1600, and umc1241. Rádi et al. (2020) employed umc1152 as a polymorphic SSR marker between donor hybrid K4390 and inducer line K405. Dong et al. (2018) screened 40 SSR primers, found four polymorphic markers (bnlg2259, bnlg2082, umc2258, umc1031) between donor ZD99 and inducer 57-1, and employed 2 of 4 markers (bnlg2259 and bnlg2082). Li et al. (2021) identified haploids using seven polymorphic SSR markers between donor ZD958 and inducer LH244.

SNP is a single base pair site in the genome that is different from one individual to another. The advantages of SNPs are, that they are abundant and widely distributed in the genome, SNP markers easy to automate, and less mutable compared to other markers such as SSRs. Prior to haploid identification using SNP genotyping, eliminating non-polymorphic markers is important. Zhao et al. (2013) screened 50,904 SNP markers using the Illumina SNP chip MaizeSNP50, and only less than 40% of those markers showed polymorphism between donor Z58 and two inducers CAU5 and CAUHOI. Kelliher et al. (2017) used Taqman zygosity assays to detect the wild-type *MTL* and mutant *mtl* alleles. Haploids had zero copies of the *mtl* allele and two copies of the maternal *MTL* allele. In contrast, diploids had one copy of the *mtl* allele and one copy of the *MTL* allele. A single polymorphic marker is ultimately sufficient for haploid identification to reduce the cost of genotyping. Multiple factors like initial assay design, the number of samples, the size of marker panels, and data turnaround time determine the final cost of genotyping per data point (1 data point = 1 sample genotyped by 1 SNP) (Semagn et al., 2014). For instance, the estimated genotyping cost can vary from US$0.10 to US$0.36 per data point and will further reduce by a certain percentage if the number of samples to be genotyped increases (Semagn et al., 2014). If 400-500 mixed seeds per induction cross are generated, the cost for haploid identification per induced ear would range between US$40 and US$50. Alternatively, SNP genotyping may serve as gold standard to confirm putative haploids previously selected via visual *R1-nj* marker, which would reduce the genotyping cost. Khammona et al. (2024) employed the *qhir1* marker to confirm putative haploid seedlings at 7 days after planting. The *qhir1* marker showed high accuracy (100%) and can be integrated in a stratified haploid identification system at early seedling stage succeeding pre-haploid sorting via *R1-nj* marker.

## Biomarkers integrated in haploid inducers

**Figure 5:**
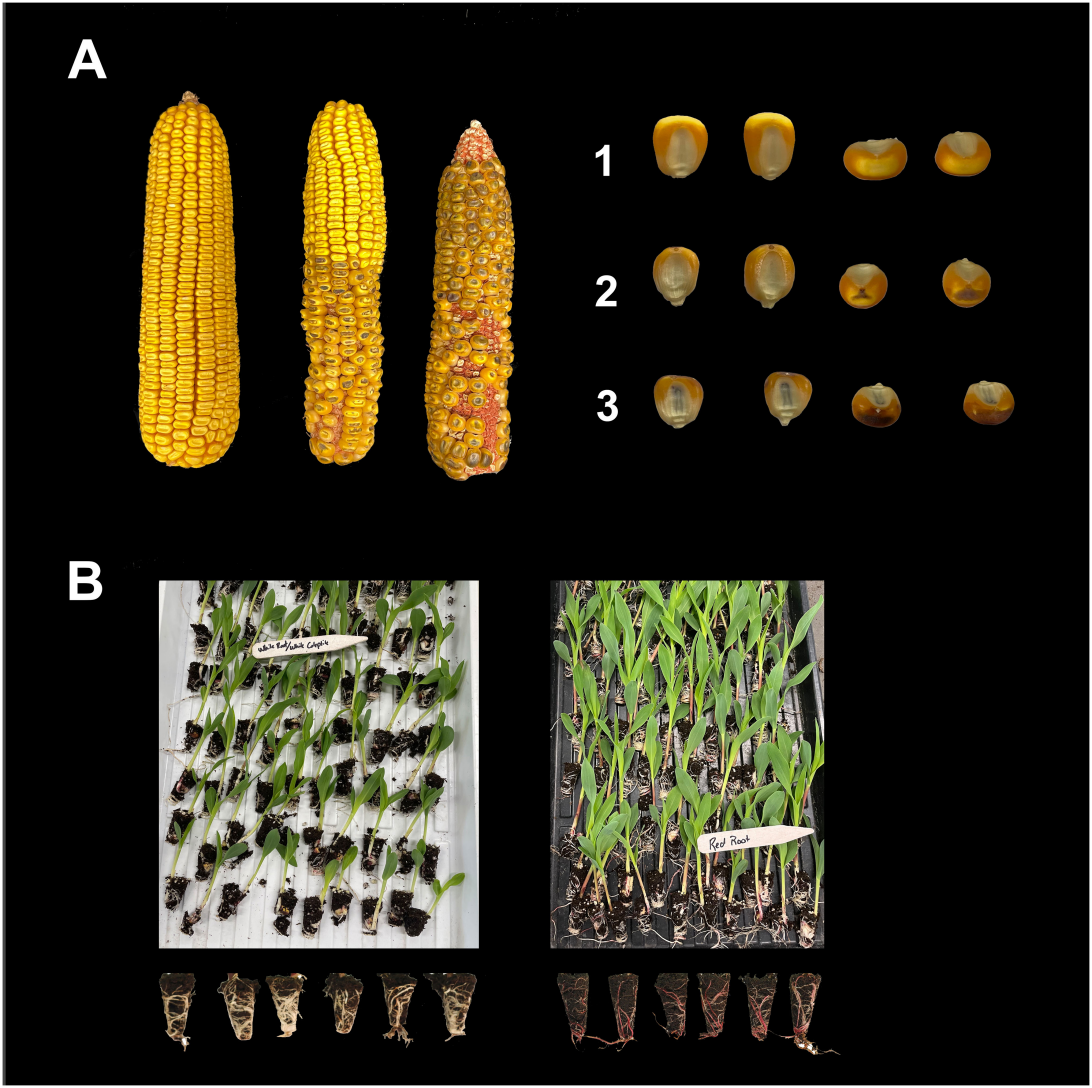
Haploid selection in maize through R1-nj kernel anthocyanin **(A)** and Pl-1 red-root **(B)** markers. A1: Outcross seeds, no R1-nj purple pigmentation in the endosperm and embryo; A2: Haploid seeds, R1-nj purple pigmentation in the endosperm but colorless embryo; A3: Diploid seeds, R1-nj purple pigmentation in the endosperm and embryo. B: White (left) and red (right) seedling roots indicate haploid and diploid seedlings, respectively.

The need for practical yet still reliable methods for haploid selection is rising when the number of samples to be sorted is large. In *in vivo* maternal DH system, one or more dominant markers are integrated in male haploid inducers while assuming that the donor females are recessive in those respective markers. This system will be effective for haploid selection only if haploid inducers are fixed in those biomarkers prior to haploid induction. For instance, in the *R1-nj* system, haploid inducers should carry homozygous dominant *R1-nj* alleles, while the *R1-nj* alleles should be absent in the donor germplasm (**Figure 5**). In this section, four biomarkers namely *R1-nj* purple kernel, *Pl-1* red root, kernel oil content, and fluorescence proteins are discussed regarding their principles of haploid (*H*) and diploid (*D*) separation, genetic properties, advantages, and limitations (**Table 2**).

**Table 2:** List of biomarkers integrated into haploid inducers for ploidy identification between haploid (H) and diploid (D) at different maize growth stages, along with their roles, genetic properties, advantages, and limitations.

Biomarker	Genetic control	Known gene or protein	Gene action	H vs. D determination	Advantages	Limitations
Navajo purple kernel ^1^	Monogenic	*R1-nj*	Additive, dominant	H: purple crown endosperm and colorless scutellum embryo. D: purple both crown endosperm and scutellum embryo.	• Early detection at the seed stage • Available in most current inducers • Practical • Non-destructive • Possible for automation	• Ineffective in source germplasm carrying *C1* anthocyanin inhibitor genes • Ineffective in source germplasm with natural anthocyanin kernels • High errors (MCR up to 80%; FDR ~ 9−43%; FNR ~ 16−28%) • Sensitive to environmental and physiological factors • Time consuming and laborious
Kernel oil content (KOC) ^2^	Polygenic	*DGAT1-2, OBAP1, WRI1*	Additive, dominant	H: lower KOC than the thresholds (*t*). D: higher KOC than the thresholds (*t*). • 3.86% vs. 5.26% • 2.6−4.3% vs. 4.6−6.1% • 2.0−4.0% vs. 5.0−6.2%	• Early detection at the seed stage • Compatible with most source germplasm • Time and labor savings • Flexible to adjust the thresholds • Could be highly accurate (± 96%) and low errors (FDR and FNR ~ <4%) • Possible for automation	• Only few inducers equipped with high KOC • Ineffective in high oil source germplasm • High investment for automation • Environmental effects • Xenia effects • Measurement errors
Fluorescence protein ^3^	–	GFP, YFP, eGFP, DsRED	Dominant	• GFP marker H: GFP negative. D: green fluorescence in emerged radicle and coleoptile. • YFP marker H: yellow signal in endosperm only. D: yellow signals in both endosperm and embryo. • DFP (eGFP + DsRED) markers H: red fluorescence in endosperm and no fluorescence in embryo. D: red fluorescence in endosperm and green in embryo.	• Robust • Early detection at the seed and seedling stages • Suitable to any source germplasm • Compatible with genome-editing platform	• Transgenic (GMO) issues • Sophisticated equipment and skilled labor for fluorescence detection
Red root ^4^	Monogenic	*Pl1*	Dominant	• H: colorless seedling roots. • D: red or purple seedling roots.	• Early detection at the seedling stage • Alternative method in progenies with either complete *R1-nj* suppression or masking *R1-nj* expression • Stratified identification methods for eliminating false positives following the *R1-nj*-based selection	• Ineffective in source germplasm with natural purple/red roots • Resource intensive for seed germinations • Light independent, which prone to increased false negatives
Purple stem, sheath, husk, and culm ^5^	Bigenic	*Pl1* and *B1*	Dominant	• H: colorless stem, sheath, husk, and culm. • D: purple stem, sheath, husk, and culm.	• Alternative method in progenies with either complete *R1-nj* suppression or masking *R1-nj* expression • Stratified identification methods for eliminating false positives following the *R1-nj*-based selection	• Incompatible with the subsequent steps in DH, which is colchicine treatments • High occurrence of natural purple sheath in source germplasm worldwide

^1^ Chase and Nanda, 1965; Nanda and Chase, 1966; Prigge et al., 2011; Chaikam et al., 2016; Melchinger et al., 2016; Dermail et al., 2023; Silva et al., 2023.

^2^ Curtis et al., 1956; Laurie et al., 2004; Wassom et al., 2008; Zheng et al., 2008; Li et al., 2009; Shen et al., 2010; Môro et al., 2012; Melchinger et al., 2013; Dong et al., 2014; López-Ribera et al., 2014; Melchinger et al., 2014; Liu et al., 2022.

^3^ Zhao et al., 2013; Yu and Birchler, 2016; Dong et al., 2018; Qi et al., 2023.

^4^ Emerson, 1921; Briggs, 1966; Coe et al., 1988; Chaikam et al., 2016; Vanous et al., 2017; Trentin et al., 2020.

^5^ Chandler et al., 1989; Coe, 1994; Chaikam et al., 2016.

As *R1-nj* and *Pl-1* biomarkers utilize anthocyanin pigmentation on different plant tissues, anthocyanin biosynthesis in maize plants is briefly highlighted. The details were described by Petroni et al. (2014). Anthocyanin biosynthesis genes in maize are regulated by two gene families, *MYB c1/pl1* and *bHLH r1/b1* (Petroni et al., 2014). The expressioN OF each member of these families is tissue- or development-specific, implying that the pattern of anthocyanin pigmentation depends on the allelic constitution at the *r1/b1* and *c1/pl1* loci. The *c1* gene in the seed or *pl1* in plant tissues contribute to the developmental and light-dependent regulation of anthocyanin accumulation, while the *bHLH* genes, such as *R1*, *B1*, *Scutellar node1 (Sn1)*, *leaf color1 (Lc1)*, *Hopi1*, determine the tissue-specific synthesis of anthocyanins (reviewed by Petroni et al., 2014). During the biosynthesis pathway of anthocyanin, multiple enzymes and genes are involved, as follows: *phenylalanine ammonia lyase* (*pal*), *cinnamic acid 4-hydroxylase* (*c4h*), *4 coumarate CoA ligase* (*4cl*), chalcone synthase (*c2*), *chalcone isomerase* (*chi1*), *flavanone 3-hydroxylase* (*f3h), flavanone 30-hydroxylase* (*pr1*), *dihydroflavonol reductase* (*a1*), *flavonol synthase* (*fls1*), *anthocyanidin synthase* (*a2*), *UDP-flavonoid glucosyl transferase* (*bz1*), *glutathione S-transferase* (*bz2*), and *anthocyanin acyltransferase* (Petroni and Tonelli, 2011; Sharma et al., 2012; Petroni et al., 2014).

### *R1-navajo* anthocyanin kernel marker

Most haploid inducer lines contain the R1-navajo *(R1-nj*), a dominant allele of the R1 gene. *R1-nj* is monogenic (Nanda and Chase, 1966) and additive inherited (Dermail et al., 2023). This marker expresses a purple pigmentation in the scutellum and aleurone layer. Post maternal haploid induction, haploid seeds are visually selected based on the *R1-nj* anthocyanin marker. Purple coloration in the aleurone layer indicates successful fertilization with the inducer line. However, it still needs to be further observed on the scutellum embryo to determine whether the seeds are haploids or diploids. Kernels with a haploid embryo (= “haploid kernels”) contain a colorless embryo and purple pigmentation on the crown of the endosperm (Chaikam et al., 2015). These haploid kernels contain triploid endosperm and haploid embryos. Diploid kernels have a purple embryo and purple endosperm. These kernels contain triploid endosperm and a diploid embryo (De La Fuente et al., 2017). This visual selection is practical since it does not require sophisticated equipment. Besides, haploid selection is not destructive and can be performed at seed stage, providing flexibility to users to arrange the timeline between haploid selection and haploid genome doubling. Those advantages make the *R1-nj* marker still the most common haploid selection method.

The selection of haploid kernels based on the *R1-nj* can be limited by factors such as (1) the presence of dominant *C1* anthocyanin inhibitor genes (*c1-I, c2-Idf, in1-D*) (Khulbe et al., 2022), (2) an occurrence of an outcross which is a result of pollen contamination from an outside source instead of an inducer line, (3) physical seed properties, (4) donor germplasm with natural anthocyanin expression in the aleurone layer, and (5) environmental factors (Prigge et al., 2011; Trentin et al., 2022).

The presence of dominant anthocyanin inhibitor genes such as *C1-I*, *C2-ldf*, and *in-1D* (Coe et al., 1988; Stinard and Sachs, 2002) suppress *R1-nj* expression. *R1-nj* suppression by*C1-I* has been reported in a large proportion of tropical maize germplasm, accounting for 25%-30% with varying suppression levels, from partial to complete inhibition (Prigge et al., 2011; Chaikam et al., 2015). The inhibitor genes may also reside in subtropical germplasm. Gain et al. (2023) noticed significant rates of R1-nj suppression in the endosperm (24%) and embryo (37%) of 178 subtropical maize inbred lines. Chaikam et al. (2015) developed a combination of two gene-specific markers (8 bp C1-I InDel and C1-I SNP) that can predict the presence of anthocyanin color inhibition in tropical germplasm with high accuracy ranging from 79% to 84%. Gain et al. (2023) also developed two C1-I specific breeder-friendly markers (MGUCI-InDel8 and MGU-C1-SNP1) that can predict the presence of the C1-I allele with 92.9% and 84.7% accuracy, respectively. Deploying those markers can assist breeders to exclude any source germplasm containing C1-I alleles from haploid induction crosses, or if those germplasms are still of importance for breeding program, those genotypes can be further purified with selection for the wild type C1 allele by discarding derivatives with the C1-I allele to enable R1-nj anthocyanin expression.

Physical seed properties such as seed shape, seed moisture content, and the presence of air pockets underneath the pericarp (Rotarenco et al., 2010; Prigge et al., 2011) can alter *R1-nj* expression. The scutellum of the embryo was more visible in flat than in round seeds, meaning that *R1-nj* expression was more intense in dent maize than in flint maize (Eder and Chalyk, 2002; Trentin et al., 2022; Thawarorit et al., 2023). The air pockets are triggered when the seed moisture content drops to low levels due to delaying harvest time and extending the duration of seed drying.

Despite being practical and cheaper than DNA markers, this method is time consuming and laborious. Our personal experience noted that one trained labor could only separate the maize seeds at the rate of six to ten ears per hour. There is also the issue of the trade-off between speed and accuracy. While increasing the speed of selection leads to higher productivity, it may come with inclined misclassification rates (MCR) as human eyes are prone to fatigue. Regarding MCR, both false positives (FDR) and false negatives (FNR) may occur simultaneously. The MCR could reach 80% with FDR ranging from 9% to 43% and FNR ranging from 16% to 28% (Melchinger et al., 2016; Silva et al., 2023). The above-mentioned limitations make haploid selection based on purple kernel markers ineffective. If high FDR is obtained, it would increase the workload in the subsequent steps such as colchicine treatments during haploid genome doubling and field transplanting. High FNR requires more haploid induction crosses to compensate for potential haploid losses.

The R locus in maize plays a crucial role in regulating the formation and distribution of anthocyanin pigment in different parts of the plant and seed. The presence of allelic variation at R locus has been reported (Styles et al., 1973), including Canada *R^g^*, Standard *R^r^*, Ecuador *R^r^*, *R^sc^*, and Cudu *R^nj^*. Those alleles obtained from various geographic sources and compared against a common genetic background exhibit a remarkable diversity in expression. For instance, Standard *R^r^* can express anthocyanin with varying levels of intensity across plant parts from low (3^rd^ internode), to moderate (coleoptile, seedling, mature plant), into high (anthers and aleurone). However, lack of expression was found in kernel embryo, making this allele unfeasible for haploid selection at seed stage. In contrast, other alleles like Canada *R^g^*, Ecuador *R^r^*, and *R^sc^* are valuable options as they express intense purple colorations on both plant and seed parts. Interestingly, the pigmentations of those alleles on the embryos are much more intense than that of Cudu *R^nj^*. In addition, Ecuador *R^r^* exhibits the earliest onset of anthocyanin pigmentation and the fastest rate of anthocyanin accumulation among others, suggesting that one may integrate Ecuador *R^r^* in haploid inducer background. It seems that there is a substantial correlation between time of onset and final concentration on anthocyanin. As each allele expresses a unique pattern, intensity, and location of anthocyanin within a plant or seed tissue, breeders can potentially utilize these genetic materials to substitute the current *R1-nj* marker. Perhaps, this could lead to more precise and effective strategies to identify haploids.

Another strategy to improve the *R1-nj* biomarker integrated in haploid inducers is to seek alternative dominant anthocyanin genes which play the same role as the *R1-nj*. Chen et al. (2022) developed two novel haploid inducers, Maize Anthocyanin Gene InduCer 1 (MAGIC1) and MAGIC2, by utilizing the co-expression of two transcription factor genes (*ZmC1* and *ZmR2*) that can activate anthocyanin biosynthesis in the embryo and aleurone layer during early seed development. MAGIC1 could identify haploids at 12 days after pollination (DAP) in most source germplasm and effectively works in germplasms carrying *C1-I*. The upgraded version, MAGIC2, could identify haploids from diploids due to differential anthocyanin accumulation in immature embryos, coleoptiles, sheaths, roots, leaves, and dry seeds. Instead of anthocyanin synthesis pathway for haploid selection, Wang et al. (2023) utilized the RUBY reporter, a betalain biosynthesis system, as a new biomarker. The expression of RUBY resulted in deep betalain pigmentation in maize embryos as early as 10 DAP and enabled 100% accuracy of immature haploid embryo identification. RUBY was also effective in germplasms carrying *C1-I* because the inhibitor *C1-I* could not prevent the synthesis of betalain.

### *Pl-1* red root marker

Given the limitations of the *R1-nj* marker, the red root marker serves as a complementary marker to the *R1-nj* in the selection of haploids. This marker is controlled by a single dominant gene, *Pl-1* (Emerson, 1921). The red root marker is an essential trait for identifying and eliminating false positives following the *R1-nj*-based selection as soon the radicle emerges (Chaikam et al., 2016; Trentin et al., 2020). In early seedling stages, the *Pl-1* gene produces red roots in diploids and colorless (white) roots in haploids (Rotarenco et al., 2010). Haploid selection based on the *Pl-1* gene at the seedling stage entails (1) germinating seeds in a growth chamber under dark conditions and at a temperature of 28 to 30°C for 72 hours, (2) maintaining the resulting seedlings for 24 hours in a dark, cold room set at 8 to 12°C to halt growth and allow anthocyanin accumulation, (3) selecting seedlings into putative diploids and putative haploids based on red and white root phenotype, respectively, and finally (4) transplanting seedlings to the field (Chaikam and Mahuku, 2012; Chaikam et al., 2016). The Doubled Haploid Facility of Iowa State University (DHF-ISU) developed Iowa Haploid Inducers (BHI), carrying the *Pl-1* as complementary markers to *R1-nj.* Initial haploid induction rates (HIR) in experiments were first calculated using the *R1-nj* anthocyanin kernel marker and corrected using the *Pl-1* red root marker (Trentin et al., 2022).

Moreover, the combination of the *Pl-1* gene with genes such as *B1* and *R1-r* produces anthocyanin pigmentation on stems, sheaths, culms, and husks of maize plants (Coe, 1994). Rotarenco et al. (2010) employed both the *B1* and *Pl-1* marker genes to develop Procera Haploid Inducer (PHI) to allow haploids to be discriminated by the lack of red expression in maize seedling roots. The PHI inducers solved the *R1-nj* marker suppression due to the inhibitor genes, and *Pl-1* was expressed as soon as four days after seed germination (Rotarenco et al., 2010). In older plants and at late seedling stage, the *Pl-1* gene produces diploid plants with purple stems and haploid plants with green stems. Thus, the *Pl-1* can be used to select haploids and diploids at various maize developmental stages.

As mentioned earlier, the adoption of *Pl-1* marker provides an alternative solution for haploid selection in progenies with either complete *R1-nj* suppression due to the inhibitor genes or masking *R1-nj* expression due to the natural purple pigmentation of source germplasm. Besides, it could reduce the false positives in progenies with partial *R1-nj* inhibition before haploid genome doubling since the red root expression can be noticed in young seedlings, seven days after sowing using plug trays (Vanous et al., 2017). This marker system has relatively lower FDRs and FNRs than the *R1-nj* marker, accounting for 13.9 vs. 24.2% (FDR) and 6.1 vs. 8.9% (FNR) (Chaikam et al., 2016). The low FDR may remain due to the slow germination of true diploids and the delay of anthocyanin accumulation in the seedling roots. The poor expression such as partial bright red and white colorations leads to human errors (Chaikam et al., 2016).

The use of the *Pl-1* gene as a complementary marker to the *R1-nj* for haploid selection could be limited by the genetic background of the source germplasm, which could produce natural red or purple pigmentation in roots. The occurrence of natural anthocyanin coloration in seedling roots of the source germplasm could increase the proportion of false negatives and makes haploid identification through red root marker ineffective (Chaikam et al., 2016). Therefore, it is imperative to evaluate the natural seedling root pigmentation of vast maize source germplasm in targeted breeding program before implementing this marker. Germinating abundant induced seeds is also resource intensive. Since the *Pl-1* gene is light-dependent (Emerson, 1921; Briggs, 1966; Coe et al., 1988), the seedling roots of some true haploids can turn red when exposed to light, making the *Pl-1*-based haploid selection prone to increased false negatives (Trentin et al., 2020). To prevent the roots from the light exposure, it is suggested to provide enough soil covering the seedlings and to keep the roots under the plug trays (Vanous et al., 2017).

### Kernel oil content

Although the complementary use *R1-nj* and *Pl-1* marker system is effective and widely adopted, it limits large-scale DH production as it is time-consuming, labor-intensive, and difficult to use with some tropical germplasm (Melchinger et al., 2016; De La Fuente et al., 2017). To overcome this limitation, a kernel oil content marker system was developed to facilitate differentiation of diploid and haploid kernels utilizing the concept of xenia effect (Alexander and Lambert, 1968; Chen and Song, 2003). Double fertilization with pollen from high-oil inducers results in viable diploid seeds showing improved KOC, while haploid seeds due to either single fertilization or post-embryo male genome elimination have low KOC and or similar KOC as source germplasm.

Four factors determine the effectiveness of KOC for haploid selection, as follows: (1) the HIR of the inducer lines; (2) the KOC difference between haploid and diploid fractions; (3) the phenotypic variance of KOC within seed fractions; and (4) the optimum thresholds of KOC values for haploid and diploid determinations (Melchinger et al., 2013). The minimum HIR should be 5%. Haploid selection will be more effective if the H vs. D difference on KOC is large, and the H vs. D overlap for KOC is small. The H vs. D difference is equal to the mid-parent value between source germplasm and inducers when assuming that KOC is additive inherited (Melchinger et al., 2013). Therefore, one could expect to gain larger H vs. D differences and smaller overlaps when applying haploid inducers with higher KOC. For instance, pollinating high oil inducers UH600 (KOC ~ 9.9%) and UH601 (KOC ~ 11.6%) to normal maize result in 1.8% of KOC difference (Melchinger et al., 2013), whereas the use of non-oil inducers such as MHI and UH400 leads to low differences, approximately 0.80% and 0.60%, respectively (Rotarenco et al., 2007; Melchinger et al., 2014). Liu et al. (2022) also compared the effectiveness of KOC marker system between oil and non-oil inducers. They noticed that non-oil inducer, CAU5 (KOC ~ 3.5%) gave high overlapping KOC distribution between haploid and diploid fractions, moderate accuracy (± 84%), and high both FDR (≥12%) and FNR (≥40%). Meanwhile, oil inducer, CHOI4 showed very little overlap between fractions, high accuracy (± 96%), and low both FDR and FNR (< 4%). The ideal HIR and KOC of haploid inducers to be compatible with diverse source germplasm should be >10% and >15%, respectively (Melchinger et al., 2013).

Breeding maize genotypes for improved KOC is doable since KOC is additive inherited (Curtis et al., 1956; Wassom et al., 2008). However, KOC is controlled by numerous quantitative trait loci (QTL). Cook et al. (2012) reported at least 20 QTL, while Laurie et al. (2004) found more than 50 QTL responsible for this trait. Among those QTL, *qHO6* is a major QTL linked to maize KOC as it encodes an acyl-CoA: diacylglycerol acyltransferase (DGAT1-2), the final catalyst for oil synthesis (Zheng et al., 2008). Regarding its polygenic control with small effects involved, KOC may be improved through long-term selection (Dudley and Lambert, 2004; Laurie et al., 2004; Song and Chen, 2004). Alexander (1988) took 27 cycles of recurrent selection to obtain 21.2% KOC in high oil maize, while Song and Chen (2004) required 18 cycles of recurrent selection to develop high KOC in Chinese oil maize from 4.7% to 15.6%. Since kernel oil is mainly stored in embryos (80-85%) (Preciado-Ortiz et al., 2013), it is suggested that breeders could perform positive selection for larger embryos to obtain high KOC. Through the Illinois Long-Term Selection Experiment, Moose et al. (2004) provided evidence that increases in embryo size led to improved KOC in high-oil Illinois maize.

Once elite maize genotypes with high KOC are obtained, those lines are intercrossed with available haploid inducers that may have high HIR but low KOC to combine and fix favorable alleles for both HIR and KOC. The China Agricultural University developed a Stock-6-derived CAUHOI inducer line with high oil content (7.8%) and HIR (2.2%) (Li et al., 2009). When crossed with the donor ZD958 (KOC ~ 3.5%), this inducer line produces high-oil content diploid kernels (6.0%) and low oil content in haploid kernels (3.8%) (Li et al., 2009). Dong et al. (2014) obtained three high oil inducer lines called CHOI1, CHOI2, and CHOI3 with average KOC of 8.5% and average HIR of 8.0% using MAS and backcrossing schemes involving inducer line CAU5 (HIR ~ 10%, KOC ~ 3.5%) and high oil inbred line GY923 (KOC ~ 10.7%) from the Alexander high oil population (Xu et al., 2013). Those high-oil-inducers produce haploid kernels with low KOC (2.6-4.3%) and diploid kernels with high KOC (4.6-6.1%), depending on donor populations used. Liu et al. (2022) developed high-oil-inducer CHOI4 (KOC ~ 11% and HIR ~ 10-21%) which was derived from founder parents between inducer line CAU2 (HIR ~ 10%) and the Beijing High Oil population (BHO) (KOC ~ 15%) (Song and Chen, 2004). The use of CHO14 as haploid inducer resulted in haploid kernels with low KOC (2.0-4.0%) and diploid kernels with high KOC (5.0-6.2%), depending on donor population used.

The KOC marker system has two main advantages over the *R1-nj* marker system including (1) flexibility to adjust the threshold for OC by considering the trade-off between FDR and FNR and (2) time and resource savings since haploid sorting via KOC can be done through automation, for instance, using NMR (Dong et al., 2014). Low thresholds for KOC result in low FDR but high FNR, meaning that users will obtain fewer false positives, but they also lose more potential true haploids. In contrast, increasing the thresholds for KOC leads to high FDR but low FNR, allowing users to gain more false positives but lose fewer true haploids (Melchinger et al., 2013). Dong et al. (2014) suggested that good thresholds should not surpass both false positives (FDR<5%) and true haploids loss (FNR<20%). The use of the KOC system still has some limitations. Environmental effects and measurement errors often cause high variation and overlapping distributions of KOC among individual diploid and haploid seeds (Melchinger et al., 2014). Besides, donor and xenia effects make breeding high oil inducers slow so that it is suggested to include at least two different testers for evaluating the HIR of haploid inducers (Dong et al., 2014).

### Fluorescence protein markers

One example in transgenic approach is the use of green fluorescent protein (GFP). The GFP gene is originally extracted from the jellyfish *Aequorea victoria*, expressing bright green fluorescence under UV light (Shimomura et al., 1962). Yu and Birchler (2016) proposed the RWS-GFP haploid inducer system to assign GFP as a dominant marker in sweet maize haploid selection. In this system, a homozygous dominant GFP transgene was inserted into a maize haploid inducer, RWS, to allow early haploid identification by visualizing the GFP expression of germinated kernels under a fluorescence microscope. Diploids will generate GFP fluorescence in emerged radicles and coleoptiles, while haploids will be GFP negative (Yu and Birchler, 2016). This approach is suitable to any maize genetic backgrounds, especially when other markers such as *R1-nj* are not available regarding a lack of *R1-nj* expression such in sweet maize (Yu and Birchler, 2016). However, a certain percentage of false positives still exists, for instance, misclassifying both diploids and aneuploids as haploids.

Cormack et al. (1996) developed several mutants of GFP (eGFP) showing 20-35 folds brighter than the wild-type GFP. It might be worth utilizing those enhanced mutants to strengthen the GFP expression. Dong et al. (2018) developed DFP-mediated haploid inducer lines (DHILs) carrying double-fluorescence protein (DFP), namely enhanced green fluorescent protein (eGFP) and the DsRED as a marker system to identify maternal haploid seeds. These dual markers have different features regarding area expression, color fluorescence, and light wavelengths. The eGFP marker expresses green embryos under 480 nm filters, while the DsRED marker expresses red endosperms under 540 nm filters. Three seed classes have different fluorescence patterns, as follows: diploids should be able to show red fluorescence from the DsRED marker in endosperm and green fluorescence from the eGFP marker in embryo; haploids show only red endosperm without green embryo; and outcross will not show any fluorescence in both areas. Qi et al. (2023) also applied the DFP markers for ploidy selection via DFP-ZC01 transgenic haploid inducers. However, they slightly modified the wavelength of filters to be 488-nm for eGFP marker and 520-nm excitation filter for DsRed2 marker.

Other fluorescence protein markers used for haploid selection are B chromosomes and centromeric histone H3-yellow fluorescent protein (CENH3-YFP). Zhao et al. (2013) used B chromosomes and CENH3-YFP for ploidy screening at kernel stage. They developed two transgenic inducers, CAU^B^ and CAU^YFP^, which carried B chromosomes and CENH3-YFP, respectively. Inducer CAU^B^ derived from crosses between inducer line CAU5 and the transgenic line B73+B, whereas inducer CAU^YFP^ derived from crosses between inducer line CAU5 and the transgenic line Hill^YFP^. Regarding the B chromosomes system, diploid cells should have 20 normal A chromosomes plus B chromosomes, while haploid cells have only 10 normal A chromosomes. It is because the B chromosomes can only be present from the male parent, which is inducer CAU^B^. Regarding the YFP system, diploid kernels express YFP signals in both endosperm and embryo, while haploid kernels show YFP signals in endosperm only. Likewise, the YFP expression can only be present from the paternal inducer CAU^YFP^. However, those two marker systems are still imperfect and can be further improved. For instance, in the B chromosomes system, there were few haploids displaying B chromosomes, confusing ploidy identification. In the YFP system, the YFP expression in the endosperm of kernels seemed to be strong only during 9 to 14 DAP, then the expression drops until 30 DAP. This system is not quite convenient in traditional DH productions as operators commonly harvest the seeds at physiological maturity (30-40 DAP). Another limitation is that it requires sophisticated equipment, such as epifluorescence microscope and CCD camera, and high skilled operators for FISH assay.

The transgenic approach via different fluorescence proteins is robust and can be integrated with genome-editing platform. However, the adoption shows slow progress due to restrictions regarding genetically modified organism (GMO) regulations. Haploids generated from the transgenic inducers are transgene-free. Dong et al. (2018) and Wang et al. (2019) independently performed Basta-resistant screening among edited haploids and confirmed that all edited haploids were Cas9-free. It implies that although transgenic haploid inducers are involved, maternal haploids generated from those inducers are still transgene-free because the paternal genome, which contains transgenes, are loss during post-fertilization in early embryo development. However, inducer chromosome segments have been found in haploids proven by morphological and molecular evidence. Some haploid kernels expressed weakly pigmented purple scutellum of embryo, high KOC, and a small proportion (± 1.84%) of the genome of paternal inducer CAUHOI (Li et al., 2009). Thus, there is a possibility that inducer transgenes can be transmitted to haploid offspring, even though the chance of this occurrence is low. However, it is sufficient for GMO regulators to not accept haploids obtained this way as non-GM.

## Automation

A commercially oriented breeding program in maize usually requires about 10,000 lines per year to evaluate, and more than half of them are DH lines (personal communication with a breeding company). If the average overall success rate (OSR), from haploid genome doubling to DH_0_ seed harvesting, is about 5-10%, at least 200,000 kernels need to be sorted. Those huge numbers often make manual sorting by either *R1-nj* or kernel oil content ineffective for commercial seed business. Since DH technology is associated with bypassing the time and resources, automation gains more attention for haploid sorting purposes. In this chapter, we emphasized automated haploid sorting at seed stage based on *R1-nj* anthocyanin and oil content. A brief comparison of each sorting approach was provided in **Table 3**.

**Table 3:** List of automated approaches for haploid sorting at kernel stage based on *R1-nj* purple and oil content markers.

Approach	Platform/instrument	Sorting time	Accuracy	Advantages	Limitations and challenges
Color optical images ^1^	A set of feeding devices, image acquisition, sorting, and control units. Image acquisition using visible RGB camera.	Up to 0.12 s per kernel.	87% − 94%	• Multiple kernels per scan. • No manual handling for positioning kernels. • Acceptable rates of seed unloading (>85%).	• a specific threshold is necessary for a specific genotype. • Unable to identify outcross seeds (colorless endosperm) from the progeny (*H* and *D*) seeds. • The accuracy claimed may be overrated.
Near-infrared spectroscopy using SIMCA analysis ^2^	• NIR spectra: A fiber-optic cable and transmitted to a Carl–Zeiss NIR spectrometer (Model MCS-611 NIR). • Algorithm for classification: Soft Independent Modeling of Class Analogy (SIMCA).	1.0-1.3 min per kernel.	85%	• Multiple kernels per scan. • No manual handling for positioning kernels.	• Further validations in a wider germplasm. • A specific spectra and model for a specific genotype. • Low throughput.
Near-infrared spectroscopy using SVSKLPP analysis ^3^	• NIR spectra: MicroNIR-1700 spectrometer. • Algorithm for classification: Supervised Virtual Sample Kernel Locality Preserving Projection (SVSKLPP).	0.016 s per kernel.	97.1%	• Multiple kernels per scan. • No manual handling for positioning kernels. • High accuracy (97.1%), sensitivity (98.8%), and specificity (95.4%). • High stability and robustness (>70% consistently for a month). • Capability to handle non-linear data. • Improving the classification effect.	• The performance of algorithm depends on the kernel matrix distance (*k*).
Fluorescence imaging ^4^	• Fluorescence spectra: A 532-nm Sapphire SF laser, a HoloSpec spectrometer, and a Newton 940 CCD camera. • Fluorescence imaging: The Typhoon FLA 9500 biomolecular imager.	12 min per 2400 kernels (0.3 s per kernel).	80% − 90%	• Multiple kernels per scan. • High selectivity and accuracy. • Acceptable misclassification rates.	• Manual handling for positioning the kernels is still required. • Thresholds in fluorescence intensity, wavelength, and area are genotype dependent.
Multispectral imaging ^5,6^	• Fluorescence and spectra: The VideometerLab 3 system with 19 different wavelengths. • Image segmentation: Canonical Discriminant Analysis (CDA).	Manual: 10-20 s per kernel. Automation: 4-5 min per 300 g kernel sample.	40% − 100%	• Multiple kernels per scan. • The instrument is well established and proven in different seed phenotyping. • Built-in software. • Compatible with the existing inducers and marker system.	• A specific wavelength for a specific genotype. • Intra-group variation increases when the non-optimum wavelength is used. • Due to an irregular kernel shape, the positioning of the kernels onto a Petri dish is not a high-throughput method. • Huge data storage from the imaging protocol (1 image ~ 1.24 Gb).
Computer vision with deep convolutional network (DeepSort) ^7^	Image-based CNN models with two convolutional layers, two densely connected layers, and an output layer.	N/A	96.8%	• The sorting output is similar to manual sorting by human experts. • Relatively robust under diverse lighting conditions, seed shapes, embryo orientation, and genetic backgrounds.	• Deep learning algorithms and modern powerful GPUs are required. • The number of layers or the depth of networks determines the sorting accuracy. • A large-scale resource for CNN models is required.
Computer vision with convolutional neural Networks (CNN) ^8,9^	Image-based CNN models with a transfer learning ^8^ and scratch ^9^ approaches.	N/A	93.4% ^8^ 94.4% ^9^	• Superior to machine learning-based methods and manual visual selection. • High selectivity and accuracy.	• A serious complexity and computational cost. • A large-scale resource for CNN models is required. • Testing with the machine for automated sorting is required. • Modern powerful GPUs are required to shorten the training times.
Kernel oil content					
Single-kernel near-infrared spectroscopy (skNIR) ^10^	• NIR spectra: skNIR platform. ^11,12^ • Algorithm: Partial least squares linear discriminant analysis (PLS-LDA).	10 kernels per second (0.1 s per kernel).	25% − 97%	• High throughput.	• Specific models for specific genotypes. • High FDR (>50%) but acceptable FNR (16%). • High accuracy depends on KOC of haploid inducers.
Near-infrared hyperspectral imaging ^13^	• NIR spectral camera: Image-λ-N17E “spectrum and image” near-infrared improved hyperspectral camera. • Algorithm: Biomimetic Uncorrelated Locality Discriminant Projection (BULDP) and biomimetic pattern recognition (BPR).	N/A	99%	• No manual handling for positioning kernels. • High accuracy to dissect *D*/*H* having overlapping oil content.	• Robustness and sorting speed is still unknown. • Further validations in a wider germplasm are required.
Time-domain nuclear magnetic resonance (TD-NMR) ^14^	• A prototype with five functional modules. • NMR measurements: a minispec mq20.	600 kernels per h (0.1 s per kernel).	>99%	• Fully automated process. • High accuracy and repeatability. • High stability and robustness. • Compatible with different NMR devices. • Sorting classes can be up to six classes. • Real-time visualization of data (histograms and summary statistics) during operation.	• High accuracy depends on KOC of haploid inducers.
NMR spectrum and manifold learning ^15^	• NMR measurements: MRI analyzer (NMI20-015V-I model). • Algorithm: single- and multiple-manifold learning.	N/A	93% − 100%	• High accuracy even when non-high oil inducers are used.	• Robustness and sorting speed is still unknown.
NMR-based method with oil content double thresholds ^16^	• NMR (model No.: Online MR20-015 V). • Two thresholds, T1 and T2, which are the upper and lower limits of the oil content, respectively.	4 s per kernel.	97.8%	• Enabling to distinguish embryo-aborted kernels from the haploids. • Reducing FDR and sorting cost.	• Sorting accuracy depends on the oil content thresholds and the xenia effects of high oil inducers.

^1^ Song et al., 2018; ^2^ Jones et al., 2012; ^3^ Yu et al., 2018; ^4^ Boote et al., 2016; ^5^ De La Fuente et al., 2017; ^6^ Videometer.com, 2023; ^7^ Veeramani et al., 2018; ^8^ Altuntaş et al., 2019; ^9^ Sabadin et al., 2021; ^10^ Gustin et al., 2020; ^11^ Armstrong and Tallada, 2012; ^12^ Spielbauer et al., 2009; ^13^ Wang et al., 2018; ^14^ Melchinger et al., 2017; ^15^ Ge et al., 2020; ^16^ Qu et al., 2021; N/A, not available.

### Kernel *R1-nj*-based automation

As mentioned earlier, manual selection with human eyes is time consuming and laborious. This method could not guarantee stable speed and accuracy since human labor is prone to fatigue against a repetitive task. Meanwhile, several approaches of *R1-nj*-based automated sorting have been proposed including machine optical sorting (Song et al., 2018), spectroscopic sorting, and computer vision methods (Veeramani et al., 2018; Altuntaş et al., 2019). For machine optical sorting, the seeds go through a hopper and the images of maize kernels are captured with a visible RGB camera to distinguish the diploid/haploid kernels based on the purple coloration in endosperm and embryo. No manual handling for positioning kernels is required as the machine is equipped with mechanical arms and solenoid valves. This platform has high accuracy (87-94%) and speed (up to 0.12 seconds per kernel) (Song et al., 2018). Since it solely relies on the optical R1-nj expressed on the surface of the kernels, higher misclassification rates may occur along the poorer expression of the R1-nj. Thus, a specific threshold is necessary for a specific donor genotype.

Spectroscopic sorting can be near-infra red (NIR) (Jones et al., 2012; Yu et al., 2018), fluorescence (Boote et al., 2016), and multispectral (De La Fuente et al., 2017). The NIR has electromagnetic spectra between visible light and mid infrared or from 780 to 2,526 nm (Pan et al., 2013). The general advantages of NIR are fast, efficient, and non-destructive (Cheng et al., 2013; Song et al., 2015). Two types of NIR spectra include reflection (Haefele et al., 2007) and transmission (Finney and Norris, 1978). Jones et al. (2012) argued that NIR transmission spectroscopy is more suitable than NIR reflection for haploid sorting because of irregular embryo surface and area marked of R1-nj expression. They applied a NIR spectroscopy with varying wavelengths, from 1,000 to 1,700 nm, and the NIR spectra were transmitted to a Carl–Zeiss NIR spectrometer (Model MCS-611 NIR). Soft Independent Modeling of Class Analogy (SIMCA) was performed as algorithms for ploidy classification according to their spectra. This platform illustrated acceptable accuracy (85%), but it can be further improved as the recent concept was low throughput (1.0-1.3 min per kernel) (Jones et al., 2012). Besides, it is genotype dependent so that specific spectra and models are still required for specific donor germplasm. Further validation in a wider germplasm is required. Zhou et al. (2007) explained that multiple factors including light, temperature, humidity, NIR intensity, instrument, and seed properties may simultaneously affect the NIR spectra of maize haploid seeds; thus, the output showed high dimensional nonlinear. To resolve that issue, Yu et al. (2018) implemented Supervised Virtual Sample Kernel Locality Preserving Projection (SVSKLPP) as nonlinear algorithms when applying NIR spectroscopy for haploid sorting. The model revealed high accuracy (97.1%), sensitivity (98.8%), and specificity (95.4%). Besides, it offers high throughput (0.016 seconds per kernel) and robustness (over 70% stable performance) for consecutive uses in a month (Yu et al., 2018).

Fluorescence imaging, a combination between fluorescence spectroscopy and imaging of maize kernels, is proposed for automated haploid sorting (Boote et al., 2016). This instrument utilizes a 532-nm Sapphire SF laser and a HoloSpec spectrometer for emitting the fluorescence spectra and Typhoon FLA 9500 biomolecular imager for imaging the fluorescence spectra. The spectra range from 550 to 700 nm, and both fluorescence intensity and spatial thresholds on fluorescence images determine the ploidy status of each sample. Haploids have larger areas of high-fluorescence intensity than diploids. The sorting speed (0.3 seconds per kernel) and accuracy (80-90%) are satisfactory (Boote et al., 2016). Multispectral imaging involving 19 different wavelengths from ultraviolet (375 nm) to NIR (970 nm) through the VideometerLab 3 system was applied to sort the haploids in maize (De La Fuente et al., 2017). This instrument is accurate (40-100%), fast (10-20 seconds per kernel), and convenient as it is well established including the software provided by third parties. Those spectral imaging methods have similar drawbacks namely (i) the needs of manual positioning the kernels for imaging and specific area and intensity thresholds for specific donor germplasm and (ii) huge data storage from the imaging protocols.

Computer vision methods with deep learning approaches are currently popular because they can learn automatically from the input/trained data (Jiang and Li, 2020). One method commonly used in deep learning is Convolutional Neural Networks (CNN). It is suitable for commercial haploid sorting as it can handle big data and simplify multiple processes between image extraction and neural network classification into a single pipeline (Ubbens and Stavness, 2017). Three models using CNN for the same sorting objective have been proposed by independent researchers (Veeramani et al., 2018; Altuntaş et al., 2019; Sabadin et al., 2021). A DeepSort CNN (Veeramani et al., 2018) illustrated 96.8% accuracy and 91.6% sensitivity. Meanwhile, Altuntaş et al. (2019) using seven CNN architectures achieved 94.2% accuracy and 94.6% sensitivity. Sabadin et al. (2021) trained the CNN model that resulted in 94.39% accuracy and 97.07% sensitivity. It seems that CNN model can be further trained to achieve optimum accuracy by adding layers of networks and enriching the input data (images) having various R1-nj expressions worldwide.

Dong et al. (2023) provided the advantages of self-supervised learning (SSL) methods in training deep learning models which often require extensive manual annotation of data. The popular self-supervised contrastive learning methods of Nearest neighbor Contrastive Learning of visual Representations (NNCLR) and Simple framework for Contrastive Learning of visual Representations (SimCLR) were implemented for the classification of spatial orientation and segmentation of embryos of maize kernels. The SSL techniques outperform their purely supervised transfer learning-based counterparts and are significantly more annotation efficient. Furthermore, a single SSL pre-trained model can be efficiently finetuned for both classification and segmentation, indicating good transferability across multiple downstream applications. They can demonstrate that SSL provides a meaningful step forward in data efficiency with agricultural deep learning and computer vision, especially for sorting haploids.

### Kernel oil content-based automation

Quantification of the kernel oil content (KOC) through analytical methods including solvent extraction, microwave-assisted extraction, and Soxtherm extraction (Kotyk et al., 2005) is destructive, time consuming, and resource intensive. Haploid seeds obtained should still be viable for subsequent steps in DH. Several non-destructive and automated methods are available for KOC-based haploid sorting, namely near-infrared (NIR) spectroscopy (Gustin et al., 2020), hyperspectral imaging (Wang et al., 2018), and nuclear magnetic resonance (Melchinger et al., 2017; Ge et al., 2020; Qu et al., 2021).

Single-kernel near-infrared reflectance spectroscopy (skNIR), a high-throughput device that captures NIR spectra ranging from 907 to 1,689 nm to predict individual kernel traits, has been applied to identify haploid kernels in maize (Gustin et al., 2020). This device utilized partial least squares linear discriminant analysis (PLS-LDA) to construct models for ploidy sorting based on skNIR data. It is high throughput (0.1 seconds per kernel), but the accuracy was inconsistent ranging from 25% to 97% depending on the KOC of haploid inducers and donor germplasm. Therefore, specific models are required for specific donor germplasm. To improve the accuracy, NIR hyperspectral imaging was implemented (Wang et al., 2018). This instrument embedded with biomimetic pattern recognition (BPR) revealed excellent accuracy (99%) even in mixed seeds with overlapping KOC between haploids and diploids.

Nuclear magnetic resonance (NMR) spectroscopy is fast, precise, and non-destructive measurement of single seeds. It is commonly applied for quantifying oil, water, protein, and hydrocarbons (Alexander et al., 1967; Rolletschek et al., 2015). Regarding NMR-based haploid sorting, different strategies to set the thresholds have been proposed. For instance, Melchinger et al. (2017) proposed time-domain NMR with five modules as a precise method for non-destructive KOC measurement. This instrument provided several advantages, as follows: (1) all steps are fully automated, (2) high accuracy and repeatability (>99%), (3) high throughput (0.1 seconds per kernel), stability, and robustness, and (4) real-time data visualization during operation (Melchinger et al., 2017). Ge et al. (2020) applied NMR with single and multiple manifold learnings for haploid sorting and achieved high accuracy (93-100%) even when non-high oil inducers are used to pollinate the donor germplasm. However, NMR with single threshold could not distinguish between haploid and embryo-aborted kernels as both fractions have lower KOC than diploid fraction, resulting in inflating false discovery rate (FDR). Therefore, NMR-based haploid sorting with KOC double thresholds is proposed (Qu et al., 2021). The upper threshold should be the minimum KOC of diploids, while the lower threshold should be the maximum oil content of embryo-aborted kernels. Applying this method, they distinguished embryo-aborted kernels from the haploids and achieved high accuracy (97.8%) and low FDR (27.9%).

### Kernel *R1-nj* and oil content-based automation

The QualySense Qsorter^®^ Explorer (QSE) is a robot that utilizes advanced mechatronics and artificial intelligence to perform single kernel sorting and analysis (Mitchell, 2023). It is considered one of the most advanced platforms for haploid sorting in the market today. The QSE is equipped with a 2D/3D camera and a high-resolution near-infrared spectrometer that enables it to sort each kernel and generate detailed reports. With the ability to inspect both physical (e.g., size, shape, color, defects) and biochemical (e.g., protein, moisture, oil, sucrose) characteristics in one pass, the QSE is a highly efficient and reliable tool for analysis.

For haploid sorting, the near-infrared spectrometer can be used to analyze compositional components, such as oil content. Additionally, the embedded 2D/3D camera can be used to sort the kernels for the presence of a purple embryo. The QSE uses multispectral imaging to examine the embryos of about 30 kernels per second, separating those with purple embryos from those with colorless embryos. It has been observed that the QSE is unable to achieve a 100% haploid recovery rate even after running a small batch twice, with the rate being less than 80%. Therefore, this robot is mainly utilized for extracting a specific number of haploids from a large batch, rather than recovering every haploid kernel within a batch. Apart from saving time, this robot has the added advantage of generating a real-time digital report that includes all the defined parameters. This feature can be incredibly useful in providing accurate and up-to-date information to the concerned parties. Another interesting feature is the flexibility to adjust the sorting thresholds of both upper and lower bounds that subsequently will determine the purity, recovery, and sorting speed. Based on the economic analysis, automated haploid sorting through the QSE is a more viable option for large breeding groups as compared to manual sorting. However, small breeding programs may not find it economically feasible to opt for the QSE.

The QSE offers a variety of tools to improve sorting accuracies, including the threshold tool, which calculates a batch’s optimal thresholds by running a sample of known haploids and diploids. The provider is constantly developing new software updates to enhance its applications, including a recent update that focuses on improving multispectral imaging in maize. This update not only improves imaging of the embryo of the kernel, but also includes the aleurone, which improves the ability to sort outcross kernels into the diploid bin. By using different cameras and camera angles to focus on more areas of the kernel, the QSE may be able to further improve sorting effectiveness.

## Outlook

Multiple tools are available in haploid identification in maize, from manual through cytological and morphological markers to high-throughput automation. Each of them plays different roles in the whole steps of haploid selection. The innate differences between haploid and diploid plants aided with heterosis phenomenon can be helpful for roguing in haploid nurseries. Cytological and molecular markers are more suitable as gold standards for haploid verification after preliminary selection via biomarkers. Breeders may favor visual haploid selection, which is practical, through biomarkers integrated in the haploid inducers. Each of the available biomarkers (*R1-nj* purple kernel, *Pl-1* red root, and kernel oil content), however, will not be effective on source germplasm which carry corresponding biomarkers. Therefore, it is important to provide haploid inducers with multiple selectable markers such as intense purple/red kernels, stem, roots, and high kernel oil content to make it compatible with any source germplasm. Automation can bypass the time and resource constraints despite high initial investment which is often unaffordable for small and medium seed enterprises.

Considering both technical and economical aspects, the ideal steps of haploid identification in maize for a commercially oriented breeding program in maize as follows: (1) preliminary identification of putative haploids via multiple biomarkers through triple options: manual, automation, or automation followed by manual; (2) haploid verification via either cytogenetics or innate differences at early seedlings (4-7 days after sowing) prior to haploid genome doubling; and (3) roguing false positives or true diploids at post haploid genome doubling in the field via innate differences at later vegetative stages. That stratified manner also acts as quality control at the haploid identification stage. Finally, increased efficiencies at the haploid identification stage can substantially increase DH line production and reduce total cost per DH line. It will lead DH technology to be feasible for maize breeding programs not only in temperate regions but also in the tropics.

## Author contributions

AD: Conceptualization, Visualization, Writing – original draft, Writing – review & editing. MM: Writing – original draft. TF: Writing – original draft. MF: Writing – original draft. Y-RC: Visualization, Writing – original draft. KS: Supervision, Writing – review & editing. UF: Conceptualization, Funding acquisition, Supervision, Writing – review & editing. TL: Conceptualization, Funding acquisition, Supervision, Writing – review & editing.
